# Supplementary material for: Identification of photoperiod-induced specific miRNAs in the adrenal glands of Sunite sheep (Ovis aries)
Source: Front Vet Sci. 2022 Jul 22;9:888207. doi: 10.3389/fvets.2022.888207 (PMC9354845; doi:10.3389/fvets.2022.888207)
Supplement: Supplementary file 1 [file Data_Sheet_1.ZIP › Supplementary file/miRDeep2 file.zip!/miRDeep2 file/Know_Structure/SPLP42A1/oar-mir-23b.pdf]

miRBase precursor : oar-mir-23b
Total read count : 15001
oar-miR-23b read count : 14975
remaining reads : 26

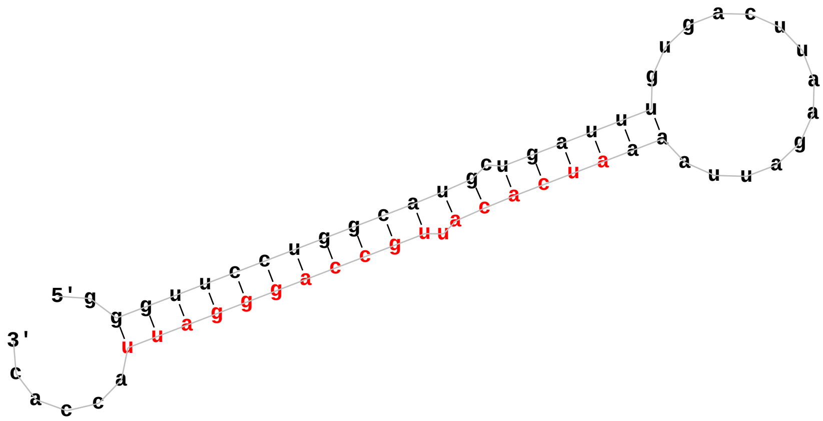

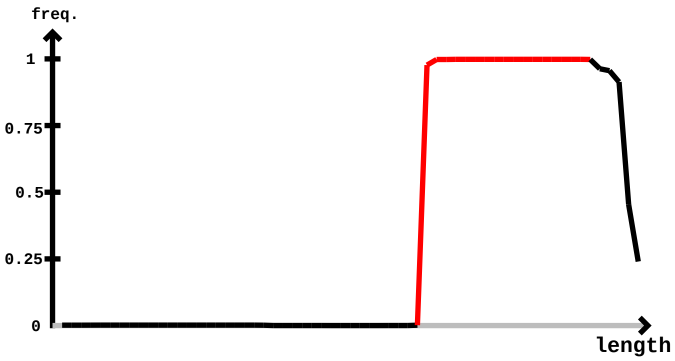


```
                                     oar-miR-23b
5'- ggguuccuggcaugcugauuugugacuuaagauuaaaaucacauugccagggauuaccac   -3'    exp
    ((((((((((((((.((((((..............)))))))).))))))))))..))..    reads      mm         sample
    ggguuccuggcaugcugauu........................................    6          0          seq
    ggguuccuggcaugcugauuu.......................................    17         0          seq
    ..guuccuggcaugcugauuuguga...................................    1          0          seq
    ...uuccuggcaugcugauuugugacu.................................    1          0          seq
    .................................uaaaaucacauugccagggauuacc..    1          0          seq
    ....................................aaucacauugccagggauu.....    2          0          seq
    ....................................aaucacauugccagggauuac...    1          0          seq
    ....................................aaucacauugccagggauuacc..    15         0          seq
    ....................................aaucacauugccagggauuacca.    2          0          seq
    .....................................aucacauugccagggau......    7          0          seq
    .....................................aucacauugccagggaC......    1          1          seq
    .....................................aucacauugcUagggauu.....    2          1          seq
    .....................................aucacauugccUgggauu.....    2          1          seq
    .....................................aucacauugccagggauu.....    516        0          seq
    .....................................aucacauugccagggauua....    107        0          seq
    .....................................aucacauugccagggauuac...    617        0          seq
    .....................................aucacauugccagggauuacc..    6780       0          seq
    .....................................aucacauugccagggauuacca.    3126       0          seq
    .....................................aucacauugccagggauuaccac    3473       0          seq
    ......................................ucacCuugccagggauu.....    2          1          seq
    ......................................ucacauugccagggauuac...    19         0          seq
    ......................................ucacauugccagggauuacc..    92         0          seq
    ......................................ucacauugccagggauuacca.    76         0          seq
    ......................................ucacauugccagggauuaccac    126        0          seq
    ........................................acauugccagggauuacc..    3          0          seq
    ........................................acauugccagggauuacca.    3          0          seq
    ........................................acauugccagggauuaccac    3          0          seq
```
